# Vitamin B12, folate and homocysteine concentrations during pregnancy and early signs of atherosclerosis at school-age

**DOI:** 10.1016/j.clnu.2021.08.001

**Published:** 2021-08-09

**Authors:** Giulietta S. Monasso, Janine F. Felix, Sandra G. Heil, Yolanda B. de Rijke, Romy Gaillard, Vincent W.V. Jaddoe

**Affiliations:** a The Generation R Study Group, Erasmus MC, University Medical Center Rotterdam, PO Box 2040, 3000, CA, Rotterdam, the Netherlands; b Department of Pediatrics, Erasmus MC, University Medical Center Rotterdam, PO Box 2040, 3000, CA, Rotterdam, the Netherlands; c Department of Clinical Chemistry, Erasmus MC, University Medical Center Rotterdam, PO Box 2040, 3000, CA, Rotterdam, the Netherlands

**Keywords:** Vitamin B12, Folate, Homocysteine, Intima-media thickness, Distensibility, Child

## Abstract

**Background & aims:**

Suboptimal circulating vitamin B12, folate and homocysteine concentrations during fetal life seem to be associated with cardiometabolic health at school-age. We examined whether fetal exposure to lower circulating vitamin B12 and folate concentrations and higher circulating homocysteine concentrations is also associated with early signs of atherosclerosis at school-age.

**Methods:**

This study among 3826 school-age children and their mothers was embedded in the Generation R Study, a population-based prospective cohort study from early pregnancy onwards. We examined the associations of early-pregnancy and cord blood serum total and active B12 and plasma folate and homocysteine concentrations with common carotid artery intima-media thickness and distensibility in the children aged ten years.

**Results:**

As compared to normal early-pregnancy serum total B12 concentrations (≥145 pmol/L), low serum total B12 concentrations (<145 pmol/L) were associated with higher carotid intima-media thickness in the children at school-age (difference 0.09 standard deviations score (SDS); 95% confidence interval (CI): 0.01, 0.16). As compared to normal early-pregnancy plasma folate concentrations (≥8 nmol/L), low plasma folate concentrations (<8 nmol/L) were associated with lower carotid disten-sibility in the children at school-age (difference −0.16 SDS; 95% CI: −0.28, −0.04). Early-pregnancy circulating total and active B12, folate and homocysteine concentrations measured continuously were not associated with carotid intima-media thickness or carotid distensibility in the children at school-age. One SDS higher plasma homocysteine concentrations measured in cord blood at birth was associated with a −0.05 SDS (95% CI: −0.09, −0.02) lower carotid distensibility at school-age. Cord blood total and active B12 and folate concentrations were not associated with carotid intima-media thickness or carotid distensibility at school-age.

**Conclusions:**

Circulating total B12, folate and homocysteine concentrations during fetal life seem to be associated with markers of subclinical atherosclerosis at school-age. Further studies need to examine the causality and mechanisms underlying these associations.

## 1 Introduction

Atherosclerosis and related cardiovascular disease seems to originate at least partly from the earliest phase of life [1–3]. Autopsy studies have shown that the formation of intimal thickening and fatty streaks, both involved in the pathogenesis of atherosclerosis, already begins in childhood and has even been observed in fetuses [4,5]. Higher carotid artery intima-media thickness and lower carotid artery distensibility are considered as early and related signs of atherosclerosis [6]. These non-invasively measured markers strongly predict future cardiovascular events in adults [7,8]. Results from observational studies suggest that higher body mass index and blood pressure, higher insulin and cholesterol concentrations, unfavorable body fat distribution, diabetes mellitus type I and parental history of premature myocardial infarction, among others, are associated with higher carotid intima-media thickness and/or impaired arterial distensibility in children [9–14]. In addition, a meta-analysis of studies assessing associations of risk factors in the first 1000 days of life with childhood carotid intima-media thickness reported that being born small for gestational age was associated with increased carotid intima-media thickness from infancy onwards [15]. This suggests that fetal nutrition and growth might be critical factors for atherogenesis.

Folate, vitamin B12 and homocysteine are micronutrients that interact in the one-carbon metabolism, which is crucial for cellular growth and differentiation, nucleic acid synthesis and DNA methylation [16,17]. Vitamin B12 and folate are important for regulating homocysteine metabolism [16,18]. In adults, higher circulating homocysteine concentrations have been associated with endothelial dysfunction, attributed to oxidative stress, and athe-rothrombotic vascular disease [17,19–21]. It is considered an independent predictor of ischemic heart disease and stroke risk [22]. Previous studies have reported associations of lower folate or vitamin B12 concentrations and higher circulating homocysteine concentrations during fetal life with a disadvantageous car-diometabolic profile at school-age [23,24].

We hypothesized that higher circulating vitamin B12 and folate concentrations and lower circulating homocysteine concentrations during fetal development are associated with higher carotid intima-media thickness and lower carotid distensibility at school-age. In a population-based study among 3826 mother–child pairs, we examined the associations of these one-carbon metabolism markers, sampled in early pregnancy and in cord blood at birth, with common carotid artery intima-media thickness and distensibility in children aged ten years.

## 2 Materials & methods

### 2.1 Design

This study was embedded in the Generation R Study, a population-based prospective cohort study from fetal life onwards in the city of Rotterdam, the Netherlands [25]. The Medical Ethical Committee of the Erasmus MC, University Medical Center in Rotterdam approved the study (MEC 198.782/2001/31). Pregnant women living in Rotterdam and with an expected delivery date between April 2002 and January 2006 were eligible to participate. Written informed consent was obtained from all participants. In the current study, we included 3826 singleton children with at least one measurement of circulating folate, vitamin B12 or homocysteine concentrations, sampled in early-pregnancy or in cord blood at birth, and information on carotid intima-media thickness or carotid distensibility measured at the median age of 9.7 years (95% range: 9.4, 10.5). Supplementary Fig. 1 shows a flowchart of the study population.

### 2.2 Total and active B12, folate and homocysteine concentrations

As described previously, maternal venous blood samples were drawn in early pregnancy (median weeks of gestation, 13.2 [Interquartile range (IQR, 12.2–14.8] weeks) and cord blood samples were taken by midwives (40.1 [IQR, 39.3–41.0] weeks gestational age) [26]. EDTA serum total B12 and serum active B12 concentrations and plasma folate and homocysteine concentrations were analyzed in the Department of Clinical Chemistry at the Erasmus MC using an electro-chemoluminescence immunoassay on the Architect System [26]. Active B12 is the active form of (total) vitamin B12 which is available to cells [27]. Active B12 concentrations were analyzed in stored serum samples only and therefore available in less participants. Reference values for deficient folate and total and active B12 status, or high homocysteine status during pregnancy are not available. We therefore applied the 95% reference intervals of healthy adults, which were established by the department of clinical chemistry [26]. Based on the lower limit of their respective 95% reference interval, we defined low/deficient (‘low’) total B12, active B12 and folate status as concentrations <145 pmol/L, <21 pmol/L and <8 nmol/L, respectively, as compared to sufficient/normal (‘normal’) concentrations. High homocysteine status was defined as concentrations above the upper limit of its 95% reference interval (≥19 μmol/L), as compared to normal concentrations. The analyses on dichotomized exposures were only possible for maternal serum total B12 and maternal plasma folate concentrations. For the other maternal and neonatal exposures, the groups with suboptimal concentrations were too small (<5% of participants). Periconceptional folic acid supplementation was assessed by questionnaires. We categorized mothers into started preconception (reference), started <10 weeks gestational age, or no supplementation.

### 2.3 Common carotid artery intima-media thickness and distensibility at school-age

When children visited the research facility at age ten years, we measured intima-media thickness and distensibility three times at both common carotid arteries (n = 5746) using the Logiq E9 (GE Medical Systems, Wauwatosa, WI, USA) device. Children were in the supine position, with the head tilted slightly away from the transducer. The common carotid artery was identified in a longitudinal plane, ~10 mm proximal from the carotid bifurcation. We obtained six recordings that ideally included multiple heart cycles. The analyses were performed offline and semi-automatically, using the application Carotid Studio (Cardiovascular Suite (Quipu srl, Pisa, Italy)). For each recording, at all R-waves of the simultaneous ECG, carotid intima-media thickness was computed at the far wall as the average distance between lumen-intima and media-adventitia borders. The average carotid intima-media thickness of all frames of the acquired image sequence was computed. The distensibility coefficient, or distensibility, was defined as the relative change in lumen area during systole for a given pressure change. Lumen diameter was computed as the average distance between the far and near media-adventitia interfaces, for each frame of the acquired image sequence. Distension was calculated as the difference between the maximal (diastolic) and minimal (systolic) lumen diameter. Per recording, the average distension and diameter values were used to compute the average carotid distensibility. During these offline analyses, we excluded 516 and 704 children, without any carotid intimamedia thickness or carotid distensibility measurement, respectively. Reasons were no appropriate recording, insufficient quality of the recording, recording of the heart only, or no blood pressure measurement available to calculate carotid distensibility. Further data processing for the remaining 5230 and 5042 children with carotid intima-media thickness and carotid distensibility data, respectively, was performed using R (R Core Team, Vienna, Austria). We excluded 9 children with unreliable low or high carotid distensibility values. All 5230 and 5033 children with at least one successful carotid intima-media thickness or carotid distensibility measurement were included in the current study. We used the overall mean carotid intima-media thickness (mm) and carotid distensibility (kPa^−1^*10^−3^) as main outcomes of interest. In a reproducibility study performed among 47 subjects, the interobserver and intraobserver intraclass correlation coefficient were greater than 0.85 for carotid distensibility and 0.94 for carotid intima-media thickness.

### 2.4 Covariates

We constructed a directed acyclic diagram (Supplementary Fig. 2). Potential covariates were selected based on previous literature, or by observing a >10% change in effect estimate, when adding the covariate to the basic model. We obtained information on maternal age, educational level according to the classification of Statistics Netherlands, pre-pregnancy body mass index, parity, smoking and alcohol consumption from questionnaires during pregnancy [25]. From midwife and hospital records we obtained information on gestational age at blood sampling, child sex and birth weight, for which we created sex- and gestational age-adjusted standard deviation scores (SDS) [28]. From questionnaires sent out at enrollment, we obtained information on child ethnicity according to the classification of Statistics Netherlands. For the current study, we re-categorized children into European and non-European ancestry. We further subdivided the children from European ancestry into Dutch ethnic ancestry, which was the largest subgroup, and non-Dutch. When children visited the research facility, we measured height and weight without shoes or heavy clothing, from which body mass index was calculated. We calculated sex- and age-adjusted SDS for body mass index based on Dutch reference growth charts (Growth Analyzer 4.0, Dutch Growth Research Foundation). We assessed blood pressure at the right brachial artery four times with 1-min intervals with the validated automatic sphygmomanometer Datascope Accutorr Plus (Paramus, New Jersey, United States). Mean systolic and diastolic blood pressure were calculated from the last three measurements [29].

### 2.5 Statistical analysis

First, we performed a non-response analysis by comparing subject characteristics with and without any carotid intima-media thickness or carotid distensibility measurement, using Student’s t-tests, Mann–Whitney tests and Chi-square tests. Second, we examined the associations of early pregnancy and cord blood total and active B12, folate and homocysteine concentrations with the overall mean carotid intima-media thickness and carotid distensibility using multivariable linear regression models. We also explored associations of folic acid supplementation and of dichotomized maternal folate and total B12 concentrations with carotid intima-media thickness and carotid distensibility. To compare effect estimates, we analyzed continuous exposures and outcomes in SDS, after natural log transformation of carotid distensibility, which had a skewed distribution. Basic models were adjusted for sex, child age and gestational age at blood sampling. Main models were additionally adjusted for maternal age, education, parity, pre-pregnancy body mass index, smoking and alcohol consumption during pregnancy and child ethnicity. We performed several sensitivity analyses. First, we explored whether birth weight SDS, and body mass index SDS and blood pressure at the same age as the carotid artery measurements substantially changed the results obtained using the main models. Birth weight and body mass index were considered as potential mediators. We considered blood pressure to be involved in the same pathway as the outcomes under investigation. Only in case of associations with P < 0.05 in the main models, we additionally adjusted for circulating concentrations of other one-carbon metabolism markers to see whether associations were specific to the respective exposure under investigation. Second, as early signs of atherosclerosis seem to depend on ethnicity, we assessed whether the effect estimates of the associations were similar in the homogeneous subgroup of children from Dutch ethnic ancestry [30]. Third, we explored the associations of all exposures with the mean intima-media thickness and distensibility of the right and left common carotid artery separately, which is recommended for carotid intima-media thickness [31]. Carotid intimamedia thickness values measured at the left side may be higher due to side-specific differences concerning blood pressure, local shear forces and vascular anatomy [32]. Because the interaction terms between maternal circulating folate and either total or active B12 concentrations were not significant (all P> 0.23), we did not further examine whether an imbalance of high folate status combined with low vitamin B12 status was associated with carotid intima-media thickness or carotid distensibility [33]. We did not stratify the analyses on sex because none of the exposures showed an interaction with sex (all P > 0.05). We observed no clear evidence for nonlinearity of the associations between any exposure and both outcomes, when we studied the exposures in quintiles, with the third quintile as the reference category. We used multiple imputations for covariates with missing values, using the Markov Chain Monte Carlo method. We created five datasets and report pooled regression coefficients [34]. All statistical analyses were performed using the Statistical Package of Social Sciences version 25.0 for Windows (SPSS IBM, Chicago, Illinois, United States).

## 3 Results

### 3.1 Subject characteristics

Table 1 and Supplementary Table 1 show subject characteristics before and after imputation of covariates, respectively. Table 2 shows the distribution of serum total and active B12 and plasma folate and homocysteine concentrations in early pregnancy and in cord blood. The same circulating one-carbon metabolism markers correlated moderately between maternal blood and cord blood (r = 0.37–0.60) (Supplementary Table 2). Circulating maternal folate and either total or active B12 concentrations were not correlated (r ≤ 0.18). The non-response analysis suggested that children without carotid intima-media thickness or carotid distensibility measurements on average had younger, more frequently lower educated and multiparous mothers, who less often drank alcohol or used folic acid supplementation, but smoked more frequently during pregnancy, and who had lower early-pregnancy circulating total and active B12 and folate concentrations (Supplementary Table 3). Furthermore, as compared to included children, those not included were less often of European ancestry, were born at slightly younger gestational age with a somewhat lower birth weight and lower cord blood folate but higher homocysteine concentrations.

### 3.2 Common carotid artery intima-media thickness and distensibility at school-age

Table 3 shows that as compared to normal maternal early-pregnancy serum total B12 concentrations (≥145 pmol/L), low total B12 concentrations (<145 pmol/L) were associated with higher carotid intima-media thickness in the children at school-age (difference 0.09 SDS; 95% confidence interval (CI): 0.01, 0.16). This association at nominal significance did not remain if we applied a Bonferroni correction, adjusting for four exposures (P <0.05/4 =0.0125). As compared to normal maternal early-pregnancy plasma folate concentrations (≥8 nmol/L), low folate concentrations (<8 nmol/L) were associated with lower carotid distensibility in the children at school-age (difference −0.16 SDS; 95% CI: −0.28, −0.04). This association did remain if we applied a Bonferroni correction. Maternal early-pregnancy total and active B12, folate and homocysteine concentrations measured continuously, and maternal folic acid supplementation during pregnancy were not associated with carotid intima-media thickness or carotid distensibility in the children at school-age (Table 3).

Cord blood total and active B12 and folate concentrations sampled at birth were not associated with carotid intima-media thickness or carotid distensibility at school-age (Table 4). However, one SDS (2.9 μmol/L) higher cord plasma homocysteine concentrations was associated with −0.05 SDS (95% CI: −0.09, −0.02) lower carotid distensibility at school-age, also after applying a Bonferroni correction. Results from the main models based on the subgroup with complete information on covariates, as well as results from basic models were largely similar (Supplementary Tables 4–6).

### 3.3 Sensitivity analyses

Supplementary Tables 7–9 show that effect estimates of all associations were largely similar in mediator models. Only the association of maternal low folate status with lower carotid distensibility in the children attenuated into non-significance after additional adjustment for child systolic blood pressure (Supplementary Table 8). The association of maternal low total B12 status with higher carotid intima-media thickness in the children, and the associations of maternal low folate status and higher cord blood homocysteine concentrations with lower carotid distensibility in the children were all not explained by other one-carbon metabolism components (Supplementary Tables 10 and 11). Supplementary Table 12 shows that results from all analyses in the homogeneous group of 2308 Dutch children were consistent with those in the full population. However, the associations we observed in the full population attenuated into non-significance, probably due to lower numbers. The results from models in which we analyzed early signs of atherosclerosis in the right and left common carotid artery separately suggested no major differences in results based on the side of carotid artery measurement (Supplementary Table 13).

## 4 Discussion

Results from our population-based prospective cohort study from early pregnancy onwards suggest that low maternal early-pregnancy total B12 concentrations are associated with higher carotid intima-media thickness in the children at school-age. In addition, low maternal early-pregnancy folate concentrations seem to be associated with lower carotid distensibility in the children at school-age. Higher cord blood homocysteine concentrations sampled at birth were associated with lower carotid distensibility at school-age.

High carotid intima-media thickness and low carotid distensibility are early signs of atherosclerosis, which may partly originate from the earliest phase of life [1,5,9–15]. Particularly during fetal development, vitamin B12 and folate are important for cellular growth and differentiation and regulating homocysteine metabolism [16,17]. Higher homocysteine concentrations have been associated with vascular disease in adults [17,19–22]. Inverse associations between circulating homocysteine concentrations and either circulating vitamin B12 or folate concentrations have been reported in relation to oxidative stress [18]. Further, suboptimal circulating vitamin B12, folate and homocysteine concentrations during fetal life seem to be associated with cardiometabolic health at school-age [23,24]. We hypothesized that higher circulating vitamin B12 and folate concentrations and lower homocysteine concentrations during fetal life are associated with more favorable carotid intima-media thickness and carotid distensibility at school-age.

We observed that low maternal early-pregnancy total B12 status was associated with higher carotid intima-media thickness in the children at school-age. Vitamin B12 is needed to convert methyltetrahydrofolate and homocysteine into tetrahydrofolate and methionine [16,17]. Tetrahydrofolate is essential for DNA synthesis whereas methionine is required for protein synthesis and ultimately for DNA methylation. Low B12 status may result in trapping of methyltetrahydrofolate and in higher homocysteine concentrations. This may compromise fetal growth and promote atherogenesis [16,17]. However, the identified association with carotid intima-media thickness seems B12-specific, as it was not explained by folate or homocysteine concentrations. We also observed that low maternal early-pregnancy folate status was associated with lower carotid distensibility in the children at school-age. Distensibility is reflective of the structural arrangement of arteries and their elastic components. Our results suggest that adaptive remodeling of the arterial wall in response to changes in blood pressure may underlie this observation [35]. This seems intuitive, as the distensibility coefficient is determined from the systolic–diastolic variations in arterial cross-sectional area and local pulse pressure [11]. Further, cord blood but not early-pregnancy homocysteine concentrations were negatively associated with carotid distensibility at school-age, suggesting a critical period in late pregnancy. Physiological changes in pregnancy or folic acid supplementation may explain lower early-pregnancy than cord blood homocysteine concentrations [36].

To the best of our knowledge, the current study is the first that assessed the associations of one-carbon metabolism markers during pregnancy with early signs of atherosclerosis in the children.

A few previous studies examined the associations of one-carbon metabolism markers with carotid intima-media thickness in adults. A study among 818 Canadians reported positive associations of circulating homocysteine but not vitamin B12 or folate concentrations with carotid intima-media thickness [37]. A study in China among 424 middle-aged siblings reported negative correlations of both circulating vitamin B12 and folate, but not of homocysteine concentrations, with carotid intima-media thickness [38]. Also, a meta-analysis of randomized controlled trials on folic acid supplementation reported no effect on carotid intima-media thickness among 1300 relatively healthy individuals [39]. Further, a review based on eight studies reported absent to weak associations of circulating homocysteine concentrations with carotid intima-media thickness [40]. Only two previous cross-sectional studies examined associations of circulating homocysteine concentrations with brachial artery distensibility in healthy individuals. Both studies, one among 383 British teenagers and another among 123 middle-aged adults, reported null findings [14,41].

The associations of one-carbon metabolism markers during fetal life with carotid intima-media thickness and carotid distensibility at school-age may be explained by the positive association between homocysteine and atherothrombogenesis [17]. This involves multiple mechanisms, all starting with the formation of reactive oxygen species [17]. Oxidative damage may result in endothelial dysfunction, proliferation of vascular smooth muscle cells, and oxidative modification of low-density lipoprotein promoting foam cell formation and lipid peroxidation, which ultimately causes degradation of nitric oxide. This vasodilator is also involved in homocysteine detoxification, which in turn may be compromised [17]. Thus, higher fetal homocysteine status may be associated with suboptimal vascular development. Alternatively, low B12 or folate concentrations may limit the conversion of homocysteine into methionine, in turn compromising the supply of methyl groups for DNA methylation [16]. Fetal DNA methylation changes may mediate associations of an adverse nutritional status *in utero* with childhood health [16]. The cyclic nature of one-carbon metabolism makes it difficult to disentangle the precise roles of vitamin B12, folate and homocysteine in the associations with subclinical atherosclerosis.

Strengths of this study are the fact that it was set in a large observational birth cohort and the availability of repeated measurements of one-carbon metabolism markers, including active B12. This study also has some weaknesses. We had no information on circulating concentrations of one-carbon metabolism markers available at school-age. The ranges of carotid intima-media thickness and carotid distensibility were small, but seem comparable with other studies among healthy children of similar age [35,42]. Importantly, previous studies reported only minimal differences between carotid intima-media thickness of children with for example obesity or diabetes mellitus, as compared to healthy children [10,12]. We also cannot exclude that rather than early structural atherosclerotic changes within the intimal layer of arteries, intima-media thickness may reflect physiological remodeling of the medial layer in response to growth [43]. Carotid intimamedia thickness and carotid distensibility measurements are subject to technical aspects, including probe resolution and image quality. As non-included children were from lower socio-economic background and exposed to less optimal one-carbon metabolism markers *in utero*, our findings may not be generalizable to the general population. We constructed a directed acyclic diagram in order to visualize relationships between exposures, outcomes and covariates. Using this approach, we attempted to minimize bias in the effect estimates of the models by selecting the relevant covariates only for inclusion in the models. Still, residual confounding might be in issue in the observed associations, as in any observational study.

## 5 Conclusion

Our findings suggest that circulating total B12, folate and homocysteine concentrations during fetal life may be associated with markers of subclinical atherosclerosis at school-age. The causality and mechanisms underlying these associations need further study.

## Supplementary Material

Supplementary data

## Figures and Tables

**Table 1:** Characteristics of participating mother—child pairs (n = 3826)^a^.

Maternal characteristics	
Age (year)	30.7 (4.8)
Educational level	
No, primary, secondary, n (%)	1818 (49.8)
Higher, n (%)	1830 (50.2)
Parity
Nulliparous, n (%)	2286 (60.0)
Multiparous, n (%)	1521 (40.0)
Pre-pregnancy body mass index (kg/m^2^)	22.6 (18.1, 34.5)
Smoking	
Non-smoker or smoked until pregnancy was known, n (%)	2901 (84.1)
Smoked throughout pregnancy, n (%)	550 (15.9)
Alcohol consumption	
Non-user or consumption until pregnancy was known, n (%)	1923 (56.4)
Sustained consumption, n (%)	1485 (43.6)
Gestational age at blood sampling (week)	13.2 (9.8, 17.4)
**Newborn characteristics**	
Gestational age (week)	40.1 (35.8, 42.3)
Birth weight (kg)	3.45 (2.22, 4.45)
Sex	
Boy, n (%)	1897 (49.6)
Girl, n (%)	1929 (50.4)
Ethnicity^b^	
European, n (%)	2583 (68.5)
Non-European, n (%)	1190 (31.5)
**Childhood characteristics**	
Age at visit (year)	9.7 (9.4, 10.5)
Common carotid artery intima-media thickness (mm)	0.46 (0.04)
Common carotid artery distensibility^c^ (kPa^-1^*10^-3^)	55.8 (37.1, 85.0)
Body mass index (kg/m^2^)	17.6 (2.8)
Blood pressure (mmHg)	
Systolic	103 (7.8)
Diastolic	59 (6.3)

a Values are based on observed, not imputed data and are mean (SD) or median (95% range) for continuous variables and numbers (%) for categorical variables.

b From questionnaires we obtained information on child ethnicity according to the classification of Statistics Netherlands. Of the children from European ethnic background, 2308 were from Dutch ethnic background and included in a sensitivity analysis.

c Indicate values before natural-log transformation.

**Table 2:** Characteristics of participants’ serum total and active B12 and plasma folate and homocysteine concentrations in early pregnancy and in cord blood sampled at birth^a,b,c^.

	Maternal early pregnancy n = 3176	Cord blood n = 2714
Serum total B12 concentration (pmol/L)	173.0 (76.0,414.0)	303.0 (120.0, 901.8)
1 SDS (pmol/L)	92.2	200.1
≥145 (pmol/L), n (%)	1972 (66.1)	2516 (94.4)
<145 (pmol/L), n (%)	1011 (33.9)	149 (5.6)
Serum active B12 concentration (pmol/L^2^)	42.0 (18.0, 98.3)	87.0 (37.0, 128.0)
1 SDS (pmol/L)	20.1	29.0
≥21 pmol/L, n (%)	2092 (95.6)	2545 (99.8)
<21 pmol/L, n (%)	96 (4.4)	6 (0.2)
Plasma folate concentration (nmol/L)	17.2 (6.0, 37.8)	20.7 (10.6, 38.5)
1 SDS (nmol/L)	9.0	7.6
≥8 nmol/L, n (%)	2795 (89.6)	2631 (99.8)
<8 nmol/L, n (%)	325 (10.4)	6 (0.2)
Plasma homocysteine concentration (μmol/L)	6.9 (4.7, 12.0)	9.0 (5.1, 16.2)
1 SDS (μmol/L)	2.0	2.9
<19 μmol/L, n (%)	3080 (99.6)	2534 (99.1)
≥19 μmol/L, n. (%)	13 (0.4)	22 (0.9)
Folic acid supplement use		
No, n (%)	622 (21.0)	–
From early pregnancy, n (%)	956 (32.3)	–
Yes, from preconception, n (%)	1385 (46.7)	–

SDS standard deviation score.

a Values are based on observed, not imputed data and are median (95% range) for continuous variables and numbers (%) for categorical variables.

b Reference values for deficient folate and total and active B12 status, or high homocysteine status during pregnancy are not available and we therefore applied the 95% reference intervals of healthy adults, which were established by the department of clinical chemistry. Table 2 shows that for early pregnancy circulating active B12 and homocysteine concentrations, and cord blood total and active B12, folate and homocysteine concentrations, the groups with suboptimal concentrations were too small for analyses.

c Maternal circulating one-carbon metabolism markers were available in n = 2983 (total B12), n = 2188 (active B12), n = 3120 (folate) and n = 3093 (homocysteine), respectively. Cord blood one-carbon metabolism markers were available in n = 2665 (total B12), n = 2551 (active B12), n = 2637 (folate) and n = 2556 (homocysteine), respectively. Information on folic acid supplement use was available in 2963 mothers with information on maternal folate concentrations in early- pregnancy.

**Table 3:** Associations of maternal circulating total and active B12, folate and homocysteine concentrations sampled in early pregnancy with early signs of atherosclerosis in children aged ten years^a,b,c^.

	Difference (95% confidence interval) in standard deviation score	
	Common carotid artery intima-media thickness^f^ n = 3826	Common carotid artery distensibility^g^ n = 3669
Total B12^d^		
Continuously, per 1 SDS	−0.02 (−0.06, 0.01)	−0.00 (−0.04, 0.04)
Dichotomous		
≥145 pmol/L	*Reference*	*Reference*
<145 pmol/L	0.09 (0.01, 0.16)*	−0.03 (−0.11, 0.04)
Active B12, continuously, per 1 SDS	−0.01 (−0.05, 0.03)	0.02 (−0.02, 0.06)
Folate^e^		
Continuously, per 1 SDS	0.01 (−0.03, 0.05)	0.00 (−0.04, 0.04)
Dichotomous		
≥8 nmol/L	*Reference*	*Reference*
<8 nmol/L	0.10 (−0.02, 0.22)	−0.16 (−0.28, −0.04)**
Homocysteine, continuously, per 1 SDS	0.01 (−0.03, 0.04)	−0.02 (−0.06, 0.01)
Folic acid supplement use		
No	*Reference*	*Reference*
From early pregnancy	0.03 (−0.08, 0.14)	0.09 (−0.02, 0.20)
From preconception	0.00 (−0.11,0.11)	0.10 (−0.01, 0.21)

SDS standard deviation score.

a Children with at least one carotid intima-media thickness or distensibility measurement available were included. Mean intima-media thickness and mean distensibility were calculated based on all available measurements of both the right and left common carotid artery. Linear regression models were adjusted for child sex, ethnicity and age at outcome; gestational age at maternal blood sampling and maternal confounders (parity, age, education, pre-pregnancy body mass index, smoking, alcohol consumption).

* P < 0.05,

** P < 0.01.

b Total and active B12 concentrations were measured in serum and folate and homocysteine concentrations were measured in plasma.

c Reference values for deficient folate and total and active B12 status, or high homocysteine status during pregnancy are not available and we therefore applied the 95% reference intervals of healthy adults, which were established by the department of clinical chemistry. Table 2 shows that for early pregnancy circulating active B12 and homocysteine concentrations, and cord blood total and active B12, folate and homocysteine concentrations, the groups with suboptimal concentrations were too small for analyses.

d Serum total B12 ≥ 145 pmol/L: n = 1,972, serum total B12 < 145 pmol/L: n = 1101.

e Plasma folate ≥8 nmol/L: n = 2,795, plasma folate <8 nmol/L: n = 325.

f Descriptive statistics of linear regression models (based on original, not imputed data): total B12 (continuous): R^2^ = 0.021, F(11, 2328) = 4.453, p < 0.001; total B12 (dichotomous): R^2^ = 0.022, F(11, 2328) = 4.780, p < 0.001; active B12: R^2^ = 0.025, F(11, 1664) = 3.817, p < 0.001; folate (continuous): R^2^ = 0.022, F(11, 2449) = 4.954, p < 0.001; folate (dichotomous): R^2^ = 0.023, F(12, 2162) = 4.228, p < 0.001; homocysteine: R^2^ = 0.022, F(11, 2426) = 4.852, p < 0.001; folic acid: R^2^ = 0.023, F(12, 2162) = 4.228, p < 0.001.

g Descriptive statistics of linear regression models (based on original, not imputed data): total B12 (continuous): R^2^ = 0.031, F(11, 2206) = 6.319, p < 0.001; total B12 (dichotomous): R^2^ = 0.031, F(11, 2206) = 6.347, p < 0.001; active B12: R^2^ = 0.020, F(11, 1637) = 2.962, p = 0.001; folate (continuous): R^2^ = 0.026, F(11, 2327) = 5.655, p < 0.001; maternal folate (dichotomous): R^2^ = 0.030, F(12, 2050) = 5.284, p < 0.001; homocysteine: R^2^ = 0.025, F(11, 2305) = 5.454, p < 0.001; folic acid: R^2^ = 0.030, F(12, 2050) = 5.284, p < 0.001.

**Table 4:** Associations of circulating total and active B12, folate and homocysteine concentrations sampled in cord blood sampled at birth with early signs of atherosclerosis in children aged ten years^a,b,c^.

	Difference (95% confidence interval) in standard deviation score	
	Common carotid artery intima-media thickness^d^ n = 3826	Common carotid artery Distensibility^e^ n = 3669
Total B12, continuously, per 1 SDS	−0.03 (−0.07, 0.01)	0.01 (−0.03, 0.05)
Active B12, continuously, per 1 SDS	0.01 (−0.03, 0.05)	0.00 (−0.04, 0.04)
Folate, continuously, per 1 SDS	−0.00 (−0.04, 0.04)	0.02 (−0.02, 0.06)
Homocysteine, continuously, per 1 SDS	−0.02 (−0.06, 0.02)	−0.05 (−0.09, −0.02)**

SDS standard deviation score.

a Children with at least one carotid intima-media thickness or carotid distensibility measurement available were included. Mean intima-media thickness and mean distensibility were calculated based on all available measurements of both the right and left common carotid artery. Linear regression models were adjusted for child sex, ethnicity and age at outcome; gestational age birth and maternal confounders (parity, age, education, pre-pregnancy body mass index, smoking, alcohol consumption).

** P < 0.01.

b Total and active B12 concentrations were measured in serum and folate and homocysteine concentrations were measured in plasma.

c Reference values for deficient folate and total and active B12 status, or high homocysteine status during pregnancy are not available and we therefore applied the 95% reference intervals of healthy adults, which were established by the department of clinical chemistry. Table 2 shows that for all neonatal exposures, the groups with sub-optimal concentrations were too small for analyses.

d Descriptive statistics of linear regression models: total B12: R^2^ = 0.023, F(11, 2071) = 4.381, p < 0.001; active B12: R^2^ = 0.023, F(11, 1984) = 4.158, p < 0.001; folate (intima-media thickness): R^2^ = 0.023, F(11, 2050) = 4.387, p < 0.001; homocysteine (intima-media thickness): R^2^ = 0.024, F(11, 1986) = 4.502, p < 0.001.

e Descriptive statistics of linear regression models: total B12: R^2^ = 0.027, F(11,1976) = 4.898, p < 0.001; active B12: R^2^ = 0.025, F(11,1892) = 4.362, p < 0.001; folate: R^2^ = 0.030, F(11,1958) = 5.458, p < 0.001; homocysteine: R^2^ = 0.032, F(11,1898) = 5.652, p < 0.001.

## Abbreviations

SDS: Standard deviation score.
