# Supplementary material for: Vitamin B12, folate and homocysteine concentrations during pregnancy and early signs of atherosclerosis at school-age
Source: Clin Nutr. Author manuscript; Available in PMC 2022 Oct 27. (PMC7613758; doi:10.1016/j.clnu.2021.08.001)
Supplement: Supplementary data [file EMS156029-supplement-Supplementary_data.docx]

**Supporting Information**

**Vitamin B12, folate and homocysteine concentrations during pregnancy and early signs of atherosclerosis at school-age**

Giulietta S. Monasso^a,b^, Janine F. Felix^a,b^, Sandra G. Heil^c^, Yolanda B. de Rijke^c^, Romy Gaillard^a,b^, Vincent W.V. Jaddoe^1,2^

1. The Generation R Study Group, Erasmus MC, University Medical Center Rotterdam, PO Box 2040, 3000 CA Rotterdam, the Netherlands
2. Department of Pediatrics, Erasmus MC, University Medical Center Rotterdam, PO Box 2040, 3000 CA Rotterdam, the Netherlands
3. Department of Clinical Chemistry, Erasmus MC, University Medical Center Rotterdam, PO Box 2040, 3000 CA Rotterdam, the Netherlands

**Corresponding Author**

Vincent W.V. Jaddoe, The Generation R Study Group (Na 29 – 08). Erasmus MC, University Medical Center Rotterdam, PO Box 2040, 3000 CA Rotterdam, the Netherlands. Phone: +31 10 7043405, Fax: +31 10 7044645, Email: v.jaddoe@erasmusmc.nl

**Number of figures: 2**

**Number of tables: 13**

**7,594** Live-born singleton children with information on at least one exposure (circulating total B12, active B12, folate and homocysteine concentrations) sampled in early pregnancy or in cord blood sampled at birth

**3,768** Excluded^a^

**2,931** Children did not visit the research center at age ten years

**587** Children who had no information on either of the outcomes

**250** Children with a (non-twin) sibling participating in the analysis

**3,826** Children included in at least one analysis

**3,176** ≥1 Exposure in early pregnancy

**2,714** ≥1 Exposure in cord blood sampled at birth

**3,826** Common carotid artery intima-media thickness

**3,532** Complete information on 6 measurements

**3,669** Common carotid artery distensibility

**2,992** Complete information on 6 measurements

**Supplementary Figure 1. Flow chart of the study population**

a The non-response analysis compared included children to those children who were not included in the analysis because they did not visit the research center (n=2,931) or had no information on either of the outcomes (n=587).

**
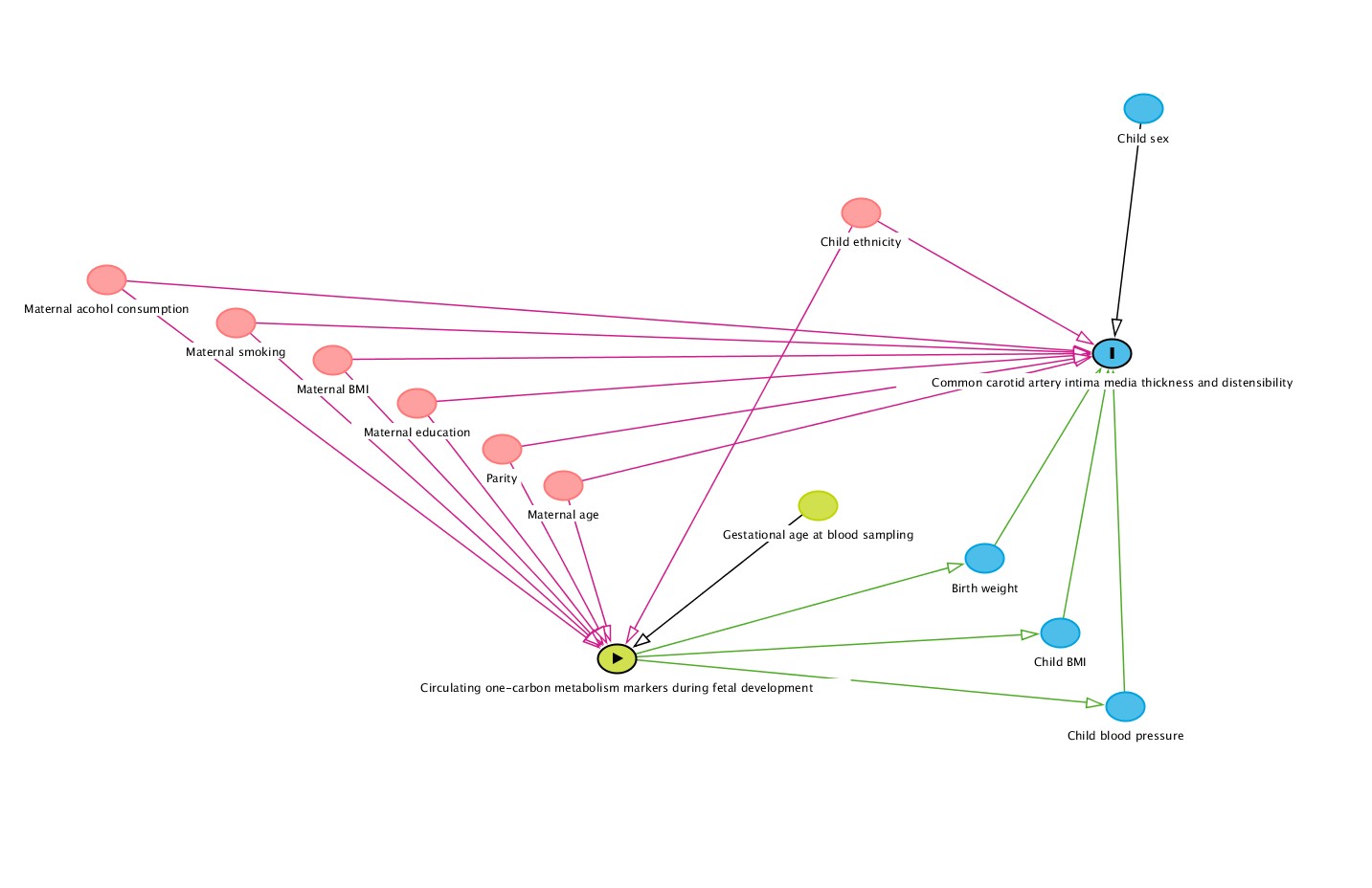
**

**Supplementary Figure 2. Directed acyclic graph**

Directed acyclic graph represents assumptions of a causal relationship between the exposures and outcomes. The other variables are considered confounders, potential mediators, or factors that influence cardio-metabolic outcomes.

**Supplementary Table 1. Characteristics of participating mother-child pairs after imputation (n=3,826)^a^**

| **Maternal characteristics** |  |
| --- | --- |
| Age (year) | 30.7 (4.8) |
| Educational level |  |
| No, primary, secondary, n (%) | 1,935 (50.6) |
| Higher, n (%) | 1,891 (49.4) |
| Parity |  |
| Nulliparous, n (%) | 2,298 (60.1) |
| Multiparous, n (%) | 1,528 (39.9) |
| Pre-pregnancy body mass index (kg/m^2^) | 22.7 (17.8, 34.1) |
| Smoking |  |
| Non-smoker or smoked until pregnancy was known, n (%) | 3,212 (84.0) |
| Smoked throughout pregnancy, n (%) | 614 (16.0) |
| Alcohol consumption |  |
| Non-user or consumption until pregnancy was known, n (%) | 2,165 (56.6) |
| Sustained consumption, n (%) | 1,661 (43.4) |
| Gestational age at blood sampling (week) | 13.3 (9.8, 17.4) |
| **Newborn characteristics** |  |
| Gestational age (week) | 40.1 (35.8, 42.3) |
| Birth weight (kg) | 3.45 (2.22, 4.45) |
| Sex |  |
| Boy, n (%) | 1,897 (49.6) |
| Girl, n (%) | 1,929 (50.4) |
| Ethnicity^b^ |  |
| European, n (%) | 2,612 (68.3) |
| Non-European, n (%) | 1,214 (31.7) |
| **Childhood characteristics** |  |
| Age at visit (year) | 9.7 (9.4, 10.5) |
| Common carotid artery intima-media thickness (mm) | 0.46 (0.04) |
| Common carotid artery distensibility^c^ (kPa^-1^*10^-3^) | 55.8 (37.1, 85.0) |
| Body mass index (kg/m^2^) | 17.6 (2.8) |
| Blood pressure (mmHg) |  |
| Systolic | 103 (7.8) |
| Diastolic | 59 (6.3) |

a Exposures and outcomes were not imputed. Values are mean (SD) or median (95% range) for continuous variables and numbers (%) for categorical variables.

b From questionnaires we obtained information on child ethnicity according to the classification of Statistics Netherlands. Of the children from European ethnic background, 2,308 were from Dutch ethnic background and included in a sensitivity analysis.

c Indicate values before natural-log transformation.

|  | **Total B12** | | **Active B12** | | **Folate** | | **Homocysteine** | |  |
| --- | --- | --- | --- | --- | --- | --- | --- | --- | --- |
|  | Maternal | Neonatal | Maternal | Neonatal | Maternal | Neonatal | Maternal | Neonatal | |
| **Total B12** |  |  |  |  |  |  |  |  | |
| Maternal | 1.00 | 0.51 | 0.67 | 0.39 | 0.15 | 0.11 | -0.24 | -0.27 | |
| Neonatal |  | 1.00 | 0.53 | 0.71 | 0.09 | 0.14 | -0.20 | -0.31 | |
| **Active B12** |  |  |  |  |  |  |  |  | |
| Maternal |  |  | 1.00 | 0.60 | 0.18 | 0.14 | -0.30 | -0.32 | |
| Neonatal |  |  |  | 1.00 | 0.07 | 0.17 | -0.20 | -0.35 | |
| **Folate** |  |  |  |  |  |  |  |  | |
| Maternal |  |  |  |  | 1.00 | 0.38 | -0.25 | -0.17 | |
| Neonatal |  |  |  |  |  | 1.00 | -0.17 | -0.29 | |
| **Homocysteine** |  |  |  |  |  |  |  |  | |
| Maternal |  |  |  |  |  |  | 1.00 | 0.37 | |
| Neonatal |  |  |  |  |  |  |  | 1.00 | |

**Supplementary Table 2. Correlation matrix of circulating vitamin 12, folate and homocysteine concentrations in early pregnancy and in cord blood sampled at birth^a,b^**

a Values represent Spearman correlation and are based on pairwise comparisons.

b Total and active B12 concentrations were measured in serum and folate and homocysteine concentrations were measured in plasma.

**Supplementary Table 3. Non-response analysis for outcome measurement in full cohort (n=7,344)^a^**

|  | **Outcome measurement**  **n=3,826** | **No outcome measurement**  **n=3,518** | **P-value^c^** |
| --- | --- | --- | --- |
| **Maternal characteristics** |  |  |  |
| Age (year) | 30.7 (4.8) | 28.5 (5.4) | <0.001 |
| Educational level |  |  | <0.001 |
| No, primary, secondary, n (%) | 1,818 (49.8) | 2,067 (66.5) |  |
| Higher, n (%) | 1,830 (50.2) | 1,043 (33.5) |  |
| Parity |  |  | <0.001 |
| Nulliparous, n (%) | 2,286 (60.0) | 1,823 (52.6) |  |
| Multiparous, n (%) | 1,521 (40.0) | 1,642 (47.4) |  |
| Pre-pregnancy body mass index (kg/m^2^) | 22.6 (18.1, 34.5) | 22.6 (17.7, 35.5) | 0.40 |
| Smoking |  |  | <0.001 |
| Non-smoker or smoked until pregnancy was known, n (%) | 2,901 (84.1) | 2,357 (77.4) |  |
| Smoked throughout pregnancy, n (%) | 550 (15.9) | 688 (22.6) |  |
| Alcohol consumption |  |  | <0.001 |
| Non-user or consumption until pregnancy was known, n (%) | 1,923 (56.4) | 2,094 (69.8) |  |
| Sustained consumption, n (%) | 1,485 (43.6) | 904 (30.2) |  |
| Gestational age at blood sampling (week) | 13.2 (9.8, 17.4) | 13.4 (9.5, 17.6) | 0.052 |
| Serum total B12 concentration (pmol/L) | 173.0 (76.0, 414.0) | 165.0 (68.0, 410.2) | <0.001 |
| Serum active B12 concentration (pmol/L) | 42.0 (18.0, 98.3) | 41.0 (17.0, 96.4) | 0.02 |
| Plasma folate concentration (nmol/L) | 17.2 (6.0, 37.8) | 13.7 (5.1, 36.8) | <0.001 |
| Plasma homocysteine concentration (μmol/L) | 6.9 (4.7, 12.0) | 7.0 (4.6, 12.4) | 0.08 |
| Folic acid supplement use ^b^ |  |  | <0.001 |
| No, n (%) | 622 (21.0) | 926 (36.3) |  |
| From early pregnancy, n (%) | 956 (32.3) | 764 (30.0) |  |
| Yes, from preconception, n (%) | 1,385 (46.7) | 858 (33.7) |  |
| **Newborn characteristics** |  |  |  |
| Gestational age, week | 40.1 (35.8, 42.3) | 40.0 (35.2, 42.3) | <0.001 |
| Birth weight, kg | 3.45 (2.22, 4.45) | 3.40 (2.15, 4.50) | <0.001 |
| Sex |  |  | 0.12 |
| Boy, n (%) | 1,897 (49.6) | 1,807 (51.4) |  |
| Girl, n (%) | 1,929 (50.4) | 1,707 (48.6) |  |
| Ethnicity |  |  | <0.001 |
| European, n (%) | 2,583 (68.5) | 1,765 (54.2) |  |
| Non-European, n (%) | 1,190 (31.5) | 1,491 (45.8) |  |
| Serum total B12 concentration (pmol/L) | 303.0 (120.0, 901.8) | 298 (118.0, 903.0) | 0.38 |
| Serum active B12 concentration (pmol/L) | 87.0 (37.0, 128.0) | 87.0 (34.0, 128.0) | 0.40 |
| Plasma folate concentration (nmol/L) | 20.7 (10.6, 38.5) | 20.1 (10.0, 38.9) | 0.004 |
| Plasma homocysteine concentration (μmol/L) | 9.0 (5.1, 16.2) | 9.2 (5.4, 17.4) | <0.001 |
| **Childhood characteristics** |  |  |  |
| Age at visit (year) | 9.7 (9.4, 10.5) | 9.8 (9.4, 12.2) | <0.001 |
| Body mass index (kg/m^2^) | 17.6 (2.8) | 17.7 (16.9) | 0.37 |

a Values are based on non-imputed data and are mean (SD) or median (95% range) for continuous variables and numbers (%) for categorical variables. The non-response analysis compared included children to those children who were not included in the analysis because they did not visit the research center (n=2,931) or had no information on both outcomes (n=587).

b Information on folic acid supplement use was available in mothers of 2,963 included and 2,548 not included children with information on maternal plasma folate concentration in early-pregnancy.

c P-values for differences in subject characteristics between groups were calculated performing independent sample t-tests (normally distributed continuous variables), Mann Whitney tests (not normally distributed continuous variables) and chi-square tests (categorical variables).

**Supplementary Table 4. Associations of maternal circulating total and active B12, folate and homocysteine concentrations sampled in early pregnancy with early signs of atherosclerosis in children aged ten years (based on observed, not imputed data in the subgroup with complete information on covariates)^a,b,c^**

|  | **Difference (95% confidence interval)**  **in standard deviation score** | |
| --- | --- | --- |
|  | **Common carotid artery intima-media thickness**  **n=2,998** | **Common carotid artery distensibility**  **n=2,862** |
| Total B12^d^ |  |  |
| Continuously, per 1 SDS | -0.01 (-0.05, 0.03) | -0.00 (-0.05, 0.04) |
| Dichotomous |  |  |
| ≥ 145 pmol/L | *Reference* | *Reference* |
| < 145 pmol/L | **0.09 (0.00, 0.17)*** | -0.03 (-0.11, 0.06) |
| Active B12, continuously, per 1 SDS | -0.01 (-0.06, 0.04) | 0.01 (-0.04, 0.06) |
| Folate^e^ |  |  |
| Continuously, per 1 SDS | -0.01 (-0.06, 0.03) | -0.01 (-0.05, 0.04) |
| Dichotomous |  |  |
| ≥ 8 nmol/L | *Reference* | *Reference* |
| < 8 nmol/L | 0.09 (-0.05, 0.22) | **-0.18 (-0.32, -0.04)*** |
| Homocysteine, continuously, per 1 SDS | 0.01 (-0.03, 0.05) | -0.01 (-0.05, 0.03) |
| Folic acid supplement use |  |  |
| No | *Reference* | *Reference* |
| From early pregnancy | 0.03 (-0.10, 0.16) | 0.10 (-0.03, 0.23) |
| From preconception | -0.01 (-0.14, 0.12) | 0.08 (-0.05, 0.21) |

SDS standard deviation score

a Children with at least one carotid intima-media thickness or carotid distensibility measurement available were included. Mean intima-media thickness and mean distensibility were calculated based on all available measurements of both the right and left common carotid artery. Linear regression models were adjusted for child sex, ethnicity and age at outcome; gestational age at maternal blood sampling and maternal confounders (parity, age, education, pre-pregnancy body mass index, smoking, alcohol consumption). *P<0.05, **P<0.01.

b Total and active B12 concentrations were measured in serum and folate and homocysteine concentrations were measured in plasma.

c Reference values for deficient folate and total and active B12 status, or high homocysteine status during pregnancy are not available and we therefore applied the 95% reference intervals of healthy adults, which were established by the department of clinical chemistry. For active B12 and homocysteine concentrations, the groups with suboptimal concentrations were too small for analyses.

d Serum total B12 ≥ 145 pmol/L: n=1,560; serum total B12 <145 pmol/L: n=780.

e Plasma folate ≥ 8 nmol/L: n=2,222; plasma folate < 8 nmol/L: n=239.

**Supplementary Table 5. Associations of circulating total and active B12, folate and homocysteine concentrations sampled in cord blood sampled at birth with early signs of atherosclerosis in children aged ten years (based on observed, not imputed data in the subgroup with complete information on covariates)^a,b,c^**

|  | **Difference (95% confidence interval) in standard deviation score** | |
| --- | --- | --- |
|  | **Common carotid artery**  **intima-media thickness**  **n=2,998** | **Common carotid artery distensibility**  **n=2,862** |
| Total B12, continuously, per 1 SDS | -0.03 (-0.08, 0.01) | -0.00 (-0.05, 0.05) |
| Active B12, continuously, per 1 SDS | 0.00 (-0.04, 0.04) | 0.02 (-0.03, 0.06) |
| Folate, continuously, per 1 SDS | -0.02 (-0.07, 0.02) | 0.02 (-0.02, 0.07) |
| Homocysteine, continuously, per 1 SDS | -0.04 (-0.08, 0.01) | **-0.05 (-0.09, -0.01)*** |

SDS standard deviation score

a Children with at least one carotid intima-media thickness or carotid distensibility measurement available were included. Mean intima-media thickness and mean distensibility were calculated based on all available measurements of both the right and left common carotid artery. Linear regression models were adjusted for child sex, ethnicity and age at outcome; gestational age birth and maternal confounders (parity, age, education, pre-pregnancy body mass index, smoking, alcohol consumption). **P<0.01.

b Total and active B12 concentrations were measured in serum and folate and homocysteine concentrations were measured in plasma.

c Reference values for deficient folate and total and active B12 status, or high homocysteine status during pregnancy are not available and we therefore applied the 95% reference intervals of healthy adults, which were established by the department of clinical chemistry. For all neonatal exposures, the groups with suboptimal concentrations were too small for analyses.

**Supplementary Table 6. Associations of circulating total and active B12, folate and homocysteine concentrations during pregnancy and in cord blood sampled at birth with early signs of atherosclerosis in children aged ten years (basic models)^a,b,c^**

|  | **Difference (95% confidence interval) in standard deviation score** | |
| --- | --- | --- |
|  | **Common carotid artery**  **intima-media thickness**  **n=3,826** | **Common carotid artery**  **distensibility**  **n=3,669** |
| **Maternal early-pregnancy** |  |  |
| Total B12 |  |  |
| Continuously, per 1 SDS | -0.02 (-0.05, 0.02) | 0.01 (-0.03, 0.05) |
| Dichotomous |  |  |
| ≥ 145 pmol/L | *Reference* | *Reference* |
| < 145 pmol/L | **0.08 (0.00, 0.15)*** | -0.05 (-0.13, 0.02) |
| Active B12, SDS | -0.00 (-0.03, 0.02) | 0.03 (0.00 0.05) |
| Folate, SDS |  |  |
| Continuously, per 1 SDS | 0.01 (-0.03, 0.04) | 0.02 (-0.02, 0.05) |
| Dichotomous |  |  |
| ≥ 8 nmol/L | *Reference* | *Reference* |
| < 8 nmol/L | 0.08 (-0.03, 0.20) | **-0.17 (-0.29, -0.06)**** |
| Homocysteine, SDS | 0.01 (-0.03, 0.04) | -0.03 (-0.07, 0.01) |
| Folic acid supplement use |  |  |
| No | *Reference* | *Reference* |
| From early pregnancy | 0.05 (-0.06, 0.16) | 0.10 (-0.01, 0.21) |
| From preconception | 0.01 (-0.09, 0.12) | **0.11 (0.00, 0.21)*** |
| **Cord blood** |  |  |
| Total B12, SDS | -0.02 (-0.06, 0.02) | 0.00 (-0.04, 0.04) |
| Active B12, SDS | 0.01 (-0.03, 0.05) | -0.00 (-0.04, 0.04) |
| Folate, SDS | -0.00 (-0.04, 0.03) | 0.02 (-0.02, 0.06) |
| Homocysteine, SDS | -0.02 (-0.06, 0.02) | **-0.06 (-0.10, -0.02)**** |

SDS standard deviation score

a Children with at least one carotid intima-media thickness or carotid distensibility measurement available were included. Mean intima-media thickness and mean distensibility were calculated based on all available measurements of both the right and left common carotid artery. Linear regression models were adjusted for child sex and age at outcome, and gestational age at blood sampling. *P<0.05, **P<0.01.

b Total and active B12 concentrations were measured in serum and folate and homocysteine concentrations were measured in plasma.

c Reference values for deficient folate and total and active B12 status, or high homocysteine status during pregnancy are not available and we therefore applied the 95% reference intervals of healthy adults, which were established by the department of clinical chemistry. For early pregnancy circulating active B12 and homocysteine concentrations, and cord blood total and active B12, folate and homocysteine concentrations, the groups with suboptimal concentrations were too small for analyses.

**Supplementary Table 7. Associations of maternal circulating total and active B12, folate and homocysteine concentrations sampled in early pregnancy with early signs of atherosclerosis in children aged ten years (mediator models)^a,b^**

|  | **Difference (95% confidence interval) in standard deviation score** | |
| --- | --- | --- |
|  | **Common carotid artery intima-media thickness**  **n=3,826** | **Common carotid artery distensibility**  **n=3,669** |
| Total B12, continuously, per 1 SDS |  |  |
| Birth weight model | -0.02 (-0.06, 0.01) | -0.00 (-0.04, 0.04) |
| Body mass index model | -0.02 (-0.06, 0.01) | 0.00 (-0.04, 0.04) |
| Systolic blood pressure model | -0.02 (-0.06, 0.01) | 0.00 (-0.03, 0.04) |
| Diastolic blood pressure model | -0.02 (-0.06, 0.01) | -0.00 (-0.04, 0.03) |
| Active B12, continuously, per 1 SDS |  |  |
| Birth weight model | -0.01 (-0.05, 0.03) | 0.02 (-0.02, 0.06) |
| Body mass index model | -0.01 (-0.06, 0.03) | 0.03 (-0.02, 0.07) |
| Systolic blood pressure model | -0.00 (-0.05, 0.04) | 0.01 (-0.03, 0.05) |
| Diastolic blood pressure model | -0.01 (-0.05, 0.03) | 0.02 (-0.02, 0.06) |
| Folate, continuously, per 1 SDS |  |  |
| Birth weight model | 0.00 (-0.03, 0.04) | 0.01 (-0.03, 0.05) |
| Body mass index model | 0.01 (-0.03, 0.05) | -0.00 (-0.04, 0.04) |
| Systolic blood pressure model | 0.01 (-0.02, 0.05) | -0.01 (-0.05, 0.03) |
| Diastolic blood pressure model | 0.01 (-0.03. 0.05) | 0.00 (-0.04, 0.04) |
| Homocysteine, continuously, per 1 SDS | 0.01 (-0.03, 0.04) | -0.03 (-0.06, 0.01) |
| Birth weight model | 0.01 (-0.02, 0.05) | -0.03 (-0.06, 0.01) |
| Body mass index model | 0.01 (-0.03, 0.04) | -0.03 (-0.06, 0.01) |
| Systolic blood pressure model | 0.01 (-0.03. 0.04) | -0.02 (-0.05, 0.02) |
| Diastolic blood pressure model | 0.01 (-0.03, 0.04) | -0.02 (-0.06, 0.01) |
| Folic acid supplement use |  |  |
| Birth weight model |  |  |
| No | *Reference* | *Reference* |
| From early pregnancy | 0.02 (-0.09, 0.13) | 0.11 (-0.00, 0.22) |
| From preconception | -0.02 (-0.13, 0.09) | **0.12 (0.00, 0.23)*** |
| Body mass index model |  |  |
| No | *Reference* | *Reference* |
| From early pregnancy | 0.04 (-0.07, 0.15) | 0.08 (-0.03, 0.19) |
| From preconception | 0.01 (-0.10, 0.12) | 0.07 (-0.05, 0.18) |
| Systolic blood pressure model |  |  |
| No | *Reference* | *Reference* |
| From early pregnancy | 0.04 (-0.07, 0.15) | 0.07 (-0.04, 0.17) |
| From preconception | 0.02 (-0.09. 0.13) | 0.05 (-0.06, 0.15) |
| Diastolic blood pressure model |  |  |
| No | *Reference* | *Reference* |
| From early pregnancy | 0.03 (-0.08, 0.14) | 0.09 (-0.02, 0.20) |
| From preconception | 0.00 (-0.11, 0.11) | 0.10 (-0.01, 0.21) |

SDS standard deviation score

a Children with at least one carotid intima-media thickness or carotid distensibility measurement available were included. Mean intima-media thickness and mean distensibility were calculated based on all available measurements of both the right and left common carotid artery. Linear regression models were adjusted for child sex, ethnicity and age at outcome; gestational age at maternal blood sampling; maternal confounders (parity, age, education, pre-pregnancy body mass index, smoking, alcohol consumption) and additionally for one of the respective potential mediators: birth weight SDS, body mass index SDS at outcome, systolic blood pressure at outcome, diastolic blood pressure at outcome. *P<0.05, **P<0.01.

b Total and active B12 concentrations were measured in serum and folate and homocysteine concentrations were measured in plasma.

**Supplementary Table 8. Associations of dichotomized maternal circulating total B12 and folate concentrations sampled in early pregnancy with early signs of atherosclerosis in children aged ten years (mediator models)^a,b,c^**

|  | **Difference (95% confidence interval) in standard deviation score** | |
| --- | --- | --- |
|  | **Common carotid artery**  **intima-media thickness**  **n=2,983** | **Common carotid artery**  **distensibility**  **n=2,982** |
| Total B12, SDS, dichotomous^d^ |  |  |
| Birth weight model |  |  |
| ≥ 145 pmol/L | *Reference* | *Reference* |
| < 145 pmol/L | **0.09 (0.01, 0.16)*** | -0.03 (-0.11, 0.04) |
| Body mass index model |  |  |
| ≥ 145 pmol/L | *Reference* | *Reference* |
| < 145 pmol/L | **0.09 (0.01, 0.16)*** | -0.04 (-0.11, 0.04) |
| Systolic blood pressure model |  |  |
| ≥ 145 pmol/L | *Reference* | *Reference* |
| < 145 pmol/L | **0.08 (0.00, 0.15)*** | -0.02 (-0.09, 0.06) |
| Diastolic blood pressure model |  |  |
| ≥ 145 pmol/L | *Reference* | *Reference* |
| < 145 pmol/L | **0.09 (0.01, 0.16)*** | -0.03 (-0.11, 0.05) |
| Folate, SDS, dichotomous^e^ |  |  |
| Birth weight model |  |  |
| ≥ 8 nmol/L | *Reference* | *Reference* |
| < 8 nmol/L | 0.12 (-0.00, 0.24) | **-0.17 (-0.29, -0.05)**** |
| Body mass index model |  |  |
| ≥ 8 nmol/L | *Reference* | *Reference* |
| < 8 nmol/L | 0.09 (-0.03, 0.20) | **-0.13 (-0.25, -0.01)*** |
| Systolic blood pressure model |  |  |
| ≥ 8 nmol/L | *Reference* | *Reference* |
| < 8 nmol/L | 0.08 (-0.04, 0.20) | -0.10 (-0.22, 0.01) |
| Diastolic blood pressure model |  |  |
| ≥ 8 nmol/L | *Reference* | *Reference* |
| < 8 nmol/L | 0.10 (-0.02, 0.22) | **-0.16 (-0.28, -0.04)**** |

SDS standard deviation score

a Children with at least one carotid intima-media thickness or carotid distensibility measurement available were included. Mean intima-media thickness and mean distensibility were calculated based on all available measurements of both the right and left common carotid artery. Linear regression models were adjusted for child sex, ethnicity and age at outcome; gestational age at maternal blood sampling; maternal confounders (parity, age, education, pre-pregnancy body mass index, smoking, alcohol consumption) and additionally for one of the respective potential mediators: birth weight SDS, body mass index SDS at outcome, systolic blood pressure at outcome, diastolic blood pressure at outcome. *P<0.05, **P<0.01. *P<0.05, **P<0.01.

b Total B12 concentrations were measured in serum and folate concentrations were measured in plasma.

c Reference values for deficient folate and total B12 status are not available and we therefore applied the 95% reference intervals of healthy adults, which were established by the department of clinical chemistry.

d Serum total B12 ≥ 145 pmol/L: n=1,972, serum total B12 <145 pmol/L: n=101.

e Plasma folate ≥ 8 nmol/L: n=2,795, plasma folate < 8 nmol/L: n=325.

**Supplementary Table 9. Associations of circulating total and active B12, folate and homocysteine concentrations sampled in cord blood sampled at birth with early signs of atherosclerosis in children aged ten years (mediator models)^a,b,c^**

|  | **Difference (95% confidence interval) in standard deviation score** | |
| --- | --- | --- |
|  | **Common carotid artery**  **intima-media thickness**  **n=3,826** | **Common carotid artery**  **distensibility**  **n=3,669** |
| Total B12, continuously, per 1 SDS |  |  |
| Birth weight model | -0.02 (-0.06, 0.02) | -0.01 (-0.05, 0.04) |
| Body mass index model | -0.03 (-0.07, 0.01) | 0.01 (-0.03, 0.05) |
| Systolic blood pressure model | -0.03 (-0.07, 0.01) | 0.01 (-0.03, 0.05) |
| Diastolic blood pressure model | -0.03 (-0.07, 0.01) | 0.00 (-0.04, 0.04) |
| Active B12, continuously, per 1 SDS |  |  |
| Birth weight model | 0.02 (-0.02, 0.06) | -0.00 (-0.04, 0.04) |
| Body mass index model | 0.01 (-0.03, 0.05) | 0.01 (-0.03. 0.05) |
| Systolic blood pressure model | 0.01 (-0.03, 0.05) | 0.00 (-0.04, 0.04) |
| Diastolic blood pressure model | 0.01 (-0.03, 0.05) | -0.00 (-0.04, 0.04) |
| Folate, continuously, per 1 SDS |  |  |
| Birth weight model | -0.01 (-0.05, 0.03) | 0.02 (-0.02, 0.06) |
| Body mass index model | -0.00 (-0.04, 0.04) | 0.02 (-0.02, 0.06) |
| Systolic blood pressure model | -0.00 (-0.04, 0.04) | 0.01 (-0.03, 0.05) |
| Diastolic blood pressure model | -0.00 (-0.04. 0.04) | 0.02 (-0.02, 0.06) |
| Homocysteine, continuously, per 1 SDS |  |  |
| Birth weight model | -0.02 (-0.06, 0.02) | **-0.05 (-0.09, -0.02)**** |
| Body mass index model | -0.02 (-0.06, 0.02) | **-0.06 (-0.10, -0.02)**** |
| Systolic blood pressure model | -0.03 (-0.06, 0.01) | **-0.05 (-0.09, -0.01)*** |
| Diastolic blood pressure model | -0.02 (-0.06, 0.02) | **-0.05 (-0.09, -0.01)*** |

SDS standard deviation score

a Children with at least one carotid intima-media thickness or carotid distensibility measurement available were included. Mean intima-media thickness and mean distensibility were calculated based on all available measurements of both the right and left common carotid artery. Linear regression models were adjusted for child sex, ethnicity and age at outcome; gestational age at birth; maternal confounders (parity, age, education, pre-pregnancy body mass index, smoking, alcohol consumption) and additionally for one of the respective potential mediators: birth weight SDS, body mass index SDS at outcome, systolic blood pressure at outcome, diastolic blood pressure at outcome. **P<0.01.

b Total and active B12 concentrations were measured in serum and folate and homocysteine concentrations were measured in plasma.

c Reference values for deficient folate and total and active B12 status, or high homocysteine status during pregnancy are not available and we therefore applied the 95% reference intervals of healthy adults, which were established by the department of clinical chemistry. For all neonatal exposures, the groups with suboptimal concentrations were too small for analyses.

**Supplementary Table 10. Associations of dichotomized maternal circulating total B12 and folate concentrations sampled in early pregnancy with early signs of atherosclerosis in children aged ten years (mutually adjusted models)^a,b,c^**

|  | **Difference (95% confidence interval) in standard deviation score** | |
| --- | --- | --- |
|  | **Common carotid artery**  **intima-media thickness**  **n=2,983** | **Common carotid artery**  **distensibility**  **n=2,982** |
| Total B12, SDS, dichotomous^d^ |  |  |
| Folate model |  | NA |
| ≥ 145 pmol/L | *Reference* |  |
| < 145 pmol/L | **0.09 (0.02, 0.17)*** |  |
| Homocysteine model |  | NA |
| ≥ 145 pmol/L | *Reference* |  |
| < 145 pmol/L | **0.09 (0.01, 0.17)*** |  |
| Folate, SDS, dichotomous^e^ |  |  |
| Total B12 model | NA |  |
| ≥ 8 nmol/L |  | *Reference* |
| < 8 nmol/L |  | **-0.15 (-0.27, -0.02)*** |
| Active B12 model | NA |  |
| ≥ 8 nmol/L |  | *Reference* |
| < 8 nmol/L |  | **-0.20 (-0.34, -0.06)**** |
| Homocysteine model | NA |  |
| ≥ 8 nmol/L |  | *Reference* |
| < 8 nmol/L |  | **-0.14 (-0.25, -0.01)*** |

SDS standard deviation score

a Children with at least one carotid intima-media thickness or carotid distensibility measurement available were included. Mean intima-media thickness and mean distensibility were calculated based on all available measurements of both the right and left common carotid artery. Linear regression models were adjusted for child sex, ethnicity and age at outcome; gestational age at maternal blood sampling; maternal confounders (parity, age, education, pre-pregnancy body mass index, smoking, alcohol consumption) and additionally for one of the respective related maternal pregnancy one-carbon metabolism marker concentrations. *P<0.05, **P<0.01. *P<0.05, **P<0.01.

b Total and active B12 concentrations were measured in serum and folate and homocysteine concentrations were measured in plasma.

c Reference values for deficient folate and total B12 status during pregnancy are not available and we therefore applied the 95% reference intervals of healthy adults, which were established by the department of clinical chemistry.

d Serum total B12 ≥ 145 pmol/L: n=1,972, serum total B12 <145 pmol/L: n=101.

e Plasma folate ≥ 8 nmol/L: n=2,795, plasma folate < 8 nmol/L: n=325.

**Supplementary Table 11. Associations of cord blood homocysteine concentrations sampled at birth with common carotid artery distensibility in children aged ten years (mutually adjusted models)^a,b^**

|  | **Difference (95% confidence interval) in standard deviation score** |
| --- | --- |
|  | **Common carotid artery distensibility**  **n=2,458** |
| Total B12 model | **-0.06 (-0.10, -0.02)**** |
| Active B12 model | **-0.06 (-0.10, -0.02)**** |
| Folate model | **-0.06 (-0.10, -0.02)**** |

SDS standard deviation score.

a Children with at least one carotid intima-media thickness or carotid distensibility measurement available were included. Mean intima-media thickness and mean distensibility were calculated based on all available measurements of both the right and left common carotid artery. Linear regression models were adjusted for child sex, ethnicity and age at outcome; gestational age at birth, child age at outcome; maternal confounders (parity, age, education, pre-pregnancy body mass index, smoking, alcohol consumption) and additionally for one of the respective related cord blood one-carbon metabolism marker concentrations. *P<0.05, **P<0.01.

b Homocysteine concentrations were measured in plasma.

**Supplementary Table 12. Associations of circulating total and active B12, folate and homocysteine concentrations during pregnancy and in cord blood sampled at birth with early signs of atherosclerosis in an ethnic homogenous group of Dutch children aged ten years (n=2,308)^a,b,c^**

|  | **Difference (95% confidence interval) in standard deviation score** | |
| --- | --- | --- |
|  | **Common carotid artery**  **intima-media thickness**  **n=2,308** | **Common carotid artery**  **distensibility**  **n=2,164** |
| **Maternal early-pregnancy** |  |  |
| Total B12 |  |  |
| Continuously, per 1 SDS | -0.02 (-0.07, 0.03) | 0.00 (-0.05, 0.05) |
| Dichotomous |  |  |
| ≥ 145 pmol/L | *Reference* | *Reference* |
| < 145 pmol/L | 0.08 (-0.02, 0.18) | -0.05 (-0.15, 0.05) |
| Active B12, SDS | -0.01 (-0.07, 0.04) | 0.03 (-0.03, 0.08) |
| Folate |  |  |
| Continuously, per 1 SDS | 0.01 (-0.04, 0.06) | 0.00 (-0.05, 0.05) |
| Dichotomous |  |  |
| ≥ 8 nmol/L | *Reference* | *Reference* |
| < 8 nmol/L | 0.10 (-0.09, 0.28) | -0.08 (-0.27, 0.11) |
| Homocysteine, SDS | 0.02 (-0.02, 0.07) | -0.02 (-0.06, 0.03) |
| Folic acid supplement use |  |  |
| No | *Reference* | *Reference* |
| From early pregnancy | 0.01 (-0.16, 0.18) | 0.03 (-0.14, 0.21) |
| From preconception | 0.02 (-0.15, 0.19) | 0.03 (-0.15, 0.20) |
| **Cord blood** |  |  |
| Total B12, SDS | -0.03 (-0.08, 0.02) | 0.03 (-0.03, 0.08) |
| Active B12, SDS | 0.01 (-0.04, 0.06) | 0.01 (-0.05, 0.06) |
| Folate, SDS | -0.04 (-0.09, 0.01) | -0.00 (-0.05, 0.05) |
| Homocysteine, SDS | 0.01 (-0.04, 0.05) | -0.04 (-0.09, 0.01) |

SDS standard deviation score

a Child ethnicity was obtained from questionnaires. Dutch children with at least one common carotid artery intima-media or distensibility measurement available were included. Mean intima-media thickness and mean distensibility were calculated based on all available measurements of both the right and left common carotid artery. Linear regression models were adjusted for child sex and age at outcome; gestational age at blood sampling and maternal confounders (parity, age, education, pre-pregnancy body mass index, smoking, alcohol consumption). *P<0.05, **P<0.01.

b Total and active B12 concentrations were measured in serum and folate and homocysteine concentrations were measured in plasma.

c Reference values for deficient folate and total and active B12 status, or high homocysteine status during pregnancy are not available and we therefore applied the 95% reference intervals of healthy adults, which were established by the department of clinical chemistry. For early pregnancy circulating active B12 and homocysteine concentrations, and cord blood total and active B12, folate and homocysteine concentrations, the groups with suboptimal concentrations were too small for analyses.

**Supplementary Table 13. Associations of circulating total and active B12, folate and homocysteine concentrations during pregnancy and in cord blood sampled at birth with early signs of atherosclerosis of the right versus left common carotid artery in children aged ten years^a,b,c^**

|  | **Difference (95% confidence interval) in standard deviation score** | | | |
| --- | --- | --- | --- | --- |
|  | **Common carotid artery**  **intima-media thickness**  **(right side)**  **n=3,773** | **Common carotid artery**  **intima-media thickness**  **(left side)**  **n=3,816** | **Common carotid artery distensibility**  **(right side)**  **n=3,627** | **Common carotid artery distensibility**  **(left side)**  **n=3,654** |
| **Maternal early-pregnancy** |  |  |  |  |
| Total B12 |  |  |  |  |
| Continuously, per 1 SDS | -0.03 (-0.06, 0.01) | -0.02 (-0.06, 0.01) | 0.01 (-0.03, 0.04) | -0.01 (-0.04, 0.03) |
| Dichotomous |  |  |  |  |
| ≥ 145 pmol/L | *Reference* | *Reference* | *Reference* | *Reference* |
| < 145 pmol/L | **0.09 (0.02, 0.17)*** | 0.06 (-0.01, 0.14) | -0.07 (-0.14, 0.01) | -0.01 (-0.09, 0.07) |
| Active B12, SDS | 0.00 (-0.04, 0.04) | -0.02 (-0.06, 0.02) | **0.05 (0.01, 0.10)*** | -0.01 (-0.05, 0.03) |
| Folate |  |  |  |  |
| Continuously, per 1 SDS | 0.02 (-0.02, 0.06) | -0.00 (-0.04, 0.04) | 0.01 (-0.03, 0.05) | -0.01 (-0.05, 0.03) |
| Dichotomous |  |  |  |  |
| ≥ 8 nmol/L | *Reference* | *Reference* | *Reference* | *Reference* |
| < 8 nmol/L | 0.09 (-0.03, 0.21) | 0.09 (-0.03, 0.21) | **-0.14 (-0.26, -0.02)*** | **-0.19 (-0.31, -0.06)**** |
| Homocysteine, SDS | 0.01 (-0.03, 0.04) | 0.01 (-0.03, 0.04) | -0.03 (-0.07, 0.01) | -0.01 (-0.05, 0.03) |
| Folic acid supplement use |  |  |  |  |
| No | *Reference* | *Reference* | *Reference* | *Reference* |
| From early pregnancy | -0.00 (-0.11, 0.11) | 0.05 (-0.06, 0.16) | 0.10 (-0.01, 0.21) | 0.05 (0.16, 0.00) |
| From preconception | 0.01 (-0.10, 0.12) | -0.01 (-0.12, 0.10) | 0.10 (-0.01, 0.22) | 0.06 (0.17, 0.00) |
| **Cord blood** |  |  |  |  |
| Total B12, SDS | -0.01 (-0.05, 0.03) | **-0.04 (-0.08, -0.00)*** | 0.02 (-0.02, 0.06) | -0.01 (-0.05, 0.03) |
| Active B12, SDS | 0.02 (-0.02, 0.06) | -0.01 (-0.04, 0.03) | 0.02 (-0.02, 0.06) | -0.02 (-0.06, 0.02) |
| Folate, SDS | 0.01 (-0.03, 0.05) | -0.02 (-0.06, 0.02) | 0.01 (-0.03, 0.05) | 0.02 (-0.02, 0.06) |
| Homocysteine, SDS | -0.03 (-0.07, 0.01) | -0.01 (-0.05, 0.03) | **-0.05 (-0.09, -0.01)*** | **-0.05 (-0.09, -0.01)*** |

SDS standard deviation score

a Children with at least one common carotid artery intima-media thickness or distensibility measurement available were included. Mean intima-media thickness and mean distensibility were calculated based on all available measurements of the right and left common carotid artery, respectively. Linear regression models were adjusted for child sex, ethnicity and age at outcome; gestational age at blood sampling and maternal confounders (parity, age, education, pre-pregnancy body mass index, smoking, alcohol consumption). *P<0.05, **P<0.01.

b Total and active B12 concentrations were measured in serum and folate and homocysteine concentrations were measured in plasma.

c Reference values for deficient folate and total and active B12 status, or high homocysteine status during pregnancy are not available and we therefore applied the 95% reference intervals of healthy adults, which were established by the department of clinical chemistry. For early pregnancy circulating active B12 and homocysteine concentrations, and cord blood total and active B12, folate and homocysteine concentrations, the groups with suboptimal concentrations were too small for analyses.
